# Exploring the Mediating Effect of Psychological Engagement on the Relationship between Child-to-Parent Violence and Violent Video Games

**DOI:** 10.3390/ijerph18062845

**Published:** 2021-03-11

**Authors:** Ana Ruiz-Fernández, Miriam Junco-Guerrero, David Cantón-Cortés

**Affiliations:** Department of Developmental and Educational Psychology, Faculty of Psychology, University of Malaga, 29013 Malaga, Spain; anruizf@hotmail.com (A.R.-F.); miriamjunco97@gmail.com (M.J.-G.)

**Keywords:** child-to-parent violence, aggression, violent video games, engagement

## Abstract

Research into the effects of violent video games on levels of aggression has raised concerns that they may pose a significant social risk, especially among younger people. The objective of this study was to analyze, through structural equation models, the mediating role of psychological engagement in the relationship between the consumption of violent video games and child-to-parent violence (CPV) against the mother and the father. The sample consisted of 916 students from the third and fourth grades of compulsory secondary education, first and second grades of high school, and first cycle of vocational training (483 males and 433 females), of whom a total of 628 were video game players, aged between 13 and 19. The exposure to video games was assessed through an author-elaborated questionnaire, engagement was evaluated with the game engagement questionnaire, and CPV was assessed through the child-to-parent aggression questionnaire. The structural equation models indicated that exposure to violent video games was related to lower rates of CPV against both parents. Conversely, the flow (a sense of being in control, being one with activity, and experiencing distortions in the perception of time) dimension of engagement positively correlated with the level of CPV against the mother, whereas the flow and absorption (total engagement in the current experience) dimensions correlated with CPV against the father. In conclusion, the results confirm the role of violent video game consumption, reducing CPV rates against both parents, a role that is offset to the extent that these violent games provoke engagement in the user.

## 1. Introduction

Child-to-parent violence (CPV) is a type of domestic violence, performed by a child or adolescent against their parents, involving physical, psychological, and/or economic harm or abuse [1]. Physical harm consists of aggressive behaviors such as hitting, pushing, or spitting, involving contact with the adult. Psychological damage refers to verbal and nonverbal behavior, in which the child intends to break the psycho-emotional balance of their parents, carrying out behaviors such as frightening the parents, intimidating or performing emotional blackmail, even threatening to commit suicide or leave home. Finally, in economic damage, the child/adolescent tries to steal money and/or belongings of value or to create debts for their parents. 

There is no scientific consensus about the incidence of CPV, although, according to studies in the legal context, it has increased in recent years. Oliva [2] reports a series of data at the national level in Spain, comparing the number of cases of assault by minors on their families in 2006 and 2010. Whereas 1627 cases of assault were recorded in 2006, 8000 assaults were reported in 2010. Additionally, some studies suggest that the same pattern is occurring in intimate partner violence [3,4]. CPV rates may increase due to the lockdown situations caused by the current COVID-19 pandemic. As the outbreak spread, nations began to shut down gatherings and confine people to their homes. As a consequence, a significant percentage of parents and practitioners have reported an increase in episodes of CPV during lockdown [5]. 

Regarding the difference between the rates of CPV types, based on adolescents’ reports, Calvete, Orue, and González [6] found that the prevalence rate ranges from 7.2 to 22% for physical aggression and from 65.8 to 93.5% for psychological aggression. On the other hand, several studies found prevalence rates according to which the violence exerted against the mother is higher than that performed against the father [7]. Moreover, previous research has shown that predictor variables of CPV towards fathers are different from predictors of CPV towards mothers [8,9,10]. Del Hoyo-Bilbao, Orue, Gámez-Guadix, and Calvete [9], for example, found that the use of corporal punishment was related to child-to-mother violence, but not CPV towards father. Conversely, Rosado and Cantón-Cortés [10] found that single-mother families, but not single-father families, had a higher rate of CPV.

The objective of this research is to analyze which variables could predict abuse against the parents, focusing on the role of the consumption of violent video games and the psychological engagement, i.e., the level of game involvement, provoked by them. The ecological perspective is a standpoint for conceptualizing the maturing person in relation to a changing psychological, physical and social environment [11]. Currently, research on CPV focuses on the antecedent psychological and social variables of CPV, such as drug consumption, the parents’ educational style, or the child’s personality and psychopathology. 

For example, numerous studies have analyzed the role of the lack of empathy, a high level of aggressive or impulsive behaviors, and egocentrism on the part of the child [8]. With regard to drug consumption, Calvete, Orue, and Gámez-Guadix [12] conducted a longitudinal study with a sample of 981 high school students. These authors found that substance abuse predicted psychological CPV both in males and females, as well as physical CPV by males. 

Concerning social variables, the effect of the peer group has been studied, finding that adolescents who have been bullied in the peer group are more likely to commit CPV [13]. Within the family sphere, the role of permissive parental style is emphasized as one of the most important predictors of CPV, as well as being a victim of abuse by the parents and a family history of marital violence [14]. 

This research focuses on the study of some variables as possible antecedents of CPV, which to date have not received attention from research on this problem, specifically the consumption of violent video games by the aggressor and the level of psychological engagement provoked by them. 

### 1.1. Video Games and Violence

During the past decade, video games have become the main industrial entertainment sector. For example, in Spain, industry data indicate that video game sales have outperformed both film and music sales [15]. Consistently, violent video games have been identified as the most popular among consumers [16,17]. As a result, research into the effects of violent video games on levels of aggression has raised concerns that they may pose a significant social risk, especially among younger people. Some authors have argued that the violence of video games and television desensitizes, and both children and adults become accustomed and familiar with it, perceiving it as normalized [18]. To date, the main theoretical framework used for the study of the relationship between violent video game consumption and aggressive behavior has been the general aggression model (GAM) [19]. In short, the GAM is a theory of social learning that proposes that repeated exposure to violence in the media promotes the accessibility of aggressive thoughts, which, in turn, increases the likelihood of cognitive schemas, emotions, and aggressive behaviors. 

Some studies have found a positive relationship between violent video game consumption and real-life aggressiveness [20,21,22,23]. DeLisi et al. [21] found that video game consumption in a sample of adolescents was associated with a higher crime rate, even after controlling for other delinquency-associated variables such as psychopathy. However, it should be noted that other investigations have found no evidence that the consumption of violent video games leads to increases in aggression or reductions in prosocial behavior [24,25] and even some studies suggest that these video games decrease players’ violence [26,27]. Cunningham et al. [26], for example, analyzed the effects of violent video game sales in the number of violent crimes, using time variation in retail unit sales data of the top 30 selling video games and violent criminal offenses both from the Uniform Crime Report and the National Incident-Based Reporting System from 2005 to 2011. Not only did these authors find no evidence of an increase in criminality associated with the consumption of violent video games, but they even found a slight decrease. 

Previous research has suggested that this disparity in results may be due, in addition to the biases of the researchers, which might resemble past moral panics with attitudes that reflect generational conflicts [28], to publication biases [29]. But these differences may also be caused by factors that mediate the relationship between violent video games and the commission of violence. Hence, variables such as the level of immersion caused by the games or addictive behaviors have been proposed [30]. Therefore, all these results indicate a clear need for further research in the area of study of violence in video games and their effects on youth. Specifically, to date, no study has sought to analyze the relationship between the use of video games and players’ commission of CPV. 

### 1.2. Violent Video Games and Engagement 

The use of video games can cause some of the players to develop high engagement in the game. This is one of the main features of video games, in which a link of engagement and participation is created between the player and the video game. For these reasons, engagement has become a considerable subject of study that requires attention [31]. According to Brockmyer et al. [30], game engagement is “a general sign of game involvement”. Engagement in video games has been considered a multidimensional concept that can be associated with some notions such as gameflow, fun, presence, immersion, or engrossment. 

Brockmyer et al. [30] proposed a model for conceptualizing engagement in video games, considering the theoretical constructs immersion, presence, flow, and absorption. These constructs can be conceptualized as a progressive increase of engagement in video games. Following Brockmyer et al. [30], immersion refers to the ability to induce the feeling of actually being part of the game or being “present” in it, although some awareness of one’s surroundings remains. Most video gamers experience some level of immersion. 

Presence is defined as having the experience of being within a virtual environment while in a normal state of consciousness. Wirth et al. [32] proposed the term ‘‘spatial presence” to describe the experience of being integrated into a mediated environment. Most, though not all, players probably have the ability to experience presence, given the appropriate conditions. 

Flow is the term used to refer to the sense of enjoyment experienced when a balance is struck between the player’s skill and the game’s challenge [31]. Flow implies a sense of being in control, being one with activity, and experiencing distortions in the perception of time. Because it involves experiencing an altered state of consciousness, the experience of flow is less common than presence or immersion.

Finally, absorption is defined as the total engagement in the current experience. As with flow, being in a state of psychological absorption induces an altered state of consciousness, causing a separation of the player’s feelings, thoughts, and experiences. Although both flow and absorption consist on states of significantly altered consciousness, flow, by definition, implies positive affect. However, absorption can also include anxiety and frustration [30].

Previous research shows that players often describe the subjective experience of playing video games with violent content as highly engaging [33]. Nowak, Krcmar, and Farrar [34] found that those players who perceived the video game as more violent experienced more presence than when they regarded it as less violent. Jeong, Biocca, and Kim [35] also analyzed the effect of violence in video games on the level of the engagement caused by them, not finding that effect in their study. However, the absence of this relationship may be because this study was based on an experimental methodology, in which the level of violence of the video game was manipulated only through the modification of the color of the blood (red to green) and the presence or absence of enemies’ cries of pain in the game. 

### 1.3. The Relationship between Engagement and Violence

Some authors have hypothesized that deep engagement in video game playing may be one of the variables that determines the effect of violent video game consumption on the player [30]. When the player is in a psychological state of absorption, there is a suspension of rational thinking, including moral assessment [36]. Brockmyer [37] also proposes that engagement arouses the development of violent behaviors because the state of engagement over time desensitizes individuals to violence, so violence would increasingly be perceived as normal. 

Farrar, Krcmar, and Nowak [38] found that, when players were more involved in a game, they experienced greater hostility and a predisposition towards physical aggression. Also, Nowak et al. [34] found that players who had high scores in presence were more verbally aggressive than those who felt lower levels of presence. High levels of presence also led to a greater trend towards physical aggression, analyzed through hypothetical situations. However, players who perceived the video game as violent, but did not experience presence, and therefore did not experience engagement, did not manifest this tendency towards hostile behaviors. 

### 1.4. Objectives

To date, numerous investigations have analyzed the effects of violent video games on general violence and, to a much lesser extent, engagement. However, no study has yet analyzed their influence on CPV. Therefore, the main objective of this study was, using structural equation models, to analyze the direct and indirect effect, through engagement, of the exposure to violent video games on CPV, towards both parents. 

The specific objectives of this study were: (1) to analyze the relationship between exposure to violence in video games and CPV against the mother and father, (2) to study the relationship between exposure to violence in video games and engagement, and (3) to analyze the relationship between engagement and CPV.

It was hypothesized that exposure to violence in video games would be associated with higher levels of CPV against the mother and father, as well as greater engagement scores. Correspondingly, engagement would be associated with higher CPV scores.

## 2. Materials and Methods

### 2.1. Participants

The sample of this study consisted of 916 participants, 483 males, and 433 females, from seven different centers of secondary education, high school, and vocational training (first middle-grade course) of southern Spain. Participants’ ages ranged from 13 to 20 years (M = 15.26, SD = 1.21), with 557 of them (60.8%) aged 13 to 15, 351 (38.3%) aged 16 to 18, and five (0.5%) aged 19 to 20. Regarding how often they play video games, 267 adolescents (29.1%) play less than once a month, 108 (11.8%) play once or twice a month, 174 (19%) play once or twice a week, 123 (13.4%) play three or four times a week, 70 (7.6%) play five or six times a week, and 153 (16.7%) play at least once a day.

For the structural equation models, only participants who had an average playing frequency of at least once a month were taken into account, and they were considered as video game players (628 adolescents of the total sample, 429 males and 199 females). The ages of the players were between 13 and 19 (M = 15.188, SD = 1.194), with 393 of them (62.5%) aged 13 to 15, 223 (35.5%) aged 16 to 17, and 10 (1.6%) aged 18 to 19. 

### 2.2. Measures

An ecological perspective was used because numerous variables may be antecedents of CPV. The effect of one of the variables most strongly associated with CPV—alcohol and drug use by participants—was therefore controlled for [39]. Concerning players’ consumption of alcohol, a total of 462 youths (73.6%) had never drunk, unlike the group of participants who drank daily, which included eight individuals (1.3%), and 155 participants (24.7%) who drank with an intermediate frequency. In terms of drug use, 579 had never used drugs (92.2%), unlike the 17 youths (2.7%) who consumed drugs every day, and 30 participants (4.8) were in the intermediate interval. Finally, most of these participants played violent video games regularly, a total of 432 youths (69.5%). The percentage of violent video game players was 81.8% for males and 42.3% for females.

Some previous studies have found effects on violence not only from video game consumption but also from exposure to violence in other media such as television [37,40,41]. Fitzpatrick, et al. [40], in their theoretical review, suggest that exposure to violence in the media, specifically television, during the preschool years represents a risk factor for the development of long-term aggressive behavior, both in childhood and adulthood. Therefore, to avoid confounding the effects of different audiovisual media, the effect of exposure to violence on television on CPV has been introduced in the model. Similarly, because previous studies have found differences between males and females in terms of CPV, the sex of the adolescents was controlled in the structural equation models [42].

### 2.3. Instruments

To collect data on the participants’ sociodemographic data, a series of questions were asked about the age, gender, and city of origin. This questionnaire also collected information about the existence of some form of alcohol and/or drug use. In the latter case, two questions were asked to determine the frequency of alcohol and drug use per week, on a five-point Likert-type scale ranging from 0 (never) to 5 (daily). Exposure to or Use of Violent Video Games. A questionnaire was designed, based on the items elaborated by Przybylski and Weinstein [43]. Participants indicate the names of the video games they have played in the last six months and the number of hours played, as well as the names of the three video games they have played the most in their lifetime. To categorize these video games as violent or non-violent, we used the classification proposed by the European PEGI (Pan European Game Information; https://pegi.info, accessed on 31 March 2020) system, a rating system used throughout most of Europe in more than 35 countries, developed by the Interactive Software Federation of Europe (ISFE). The criteria followed to consider a participant as a violent video game player were as follows: (1) one of the video games described as the one played the most in their life is either a video game suitable for over-18-year-olds or two video games suitable for over-16-year-olds, or (2) participants indicate that they have played violent video games for more than 50 h in the last six months, taking into account the age rating of the games (multiplying by 1) the number of hours in the case of games suitable for over-16-year-olds, and by 2) if the game is suitable for over-18-year-olds). Game Engagement Questionnaire (GEQ; [30]). The questionnaire has a total of 19 items, which measure four different types of engagement: presence (four items, e.g., “I lose the notion of time”), immersion (one item, e.g., “I feel like I can’t stop playing”), Flow (nine items, e.g., “if someone speaks to me, I do not hear him”), and absorption (five items, e.g., “I lose track of where I am”). Participants indicate which of these events occurred when they played video games. Each item is rated on a three-point Likert-type scale (1 = it doesn’t happen to me; 2 = it happens to me a little bit; 3 = it happens to me a lot). In this study, this instrument obtained a global Cronbach alpha internal consistency coefficient for video games players of 0.81, that is, 0.68 for the presence subscale, 0.73 for the flow subscale, and 0.72 for the absorption subscale. Cuestionario de Exposición a la Violencia (CEV, Exposure to Violence Questionnaire [13]). This questionnaire has a total of 21 items, which evaluate exposure to violent behaviors in different contexts: at the school (six items), on the street (six items), at home (six items), and on television (three items). Participants indicate the frequency with which violent actions have occurred in the different contexts. Each item is scored on a five-point Likert-type scale ranging from 0 (never) to 4 (every day). In this case, only scores for television violence exposure (e.g., “How often have you seen a person insult someone on TV?”) were taken into account. In this study, Cronbach’s alpha internal consistency coefficient for the television violence exposure scale was 0.81. Child-to-Parent Aggression Questionnaire (CPAQ; [44]). This instrument assesses violence performed by children and youths against their parents. It consists of 20 parallel items: 10 referring to the mother, and 10 to the father. In each block of 10 items, seven of them refer to psychological violence (e.g., “You yelled at your mother/father when you were angry”), and three to physical aggressions (e.g., “You have pushed or hit your mother/father in a fight”). In addition to the items in the original questionnaire, an item was included to assess financial violence (“You have taken money from your father/mother without permission”). Participants indicate how often they have committed these types of aggressions against their parents in the past year on a Likert scale: 0 (never, this has not happened in my relationship with my mother or father), 1 (this has happened rarely, one or two times), 2 (sometimes, it has occurred between three and five times), and 3 (very often, it has occurred six times or more). In our study, Cronbach’s alpha coefficient was 0.69 and 0.71 for physical violence against mothers and fathers, and 0.74 and 0.71 for psychological violence against mothers and fathers, respectively.

### 2.4. Procedure

First, the approval of the Ethical Committee of Experimentation of the University of Malaga was obtained, with registration number 44-2020-H. Permission was sought from the different schools in order to administer the survey within them. Thus, at each school, the first contact was held with the School Board and the Department of Educational Guidance, explaining the nature and objective of the research to obtain their consent. All the centers we contacted were willing to participate in the research. The questionnaire was applied in seven public schools in southern Spain.

The participants were requested to give their informed consent and were informed that the completion of the questionnaire was strictly confidential and voluntary, so none of them should specify data that could identify them. All of the students who were requested to participate were willing to do so. Parents were notified and given the option of refusing to allow their child’s participation. None of the parents refused to allow their child to participate. The administration of the questionnaire was carried out in groups, in school classrooms or the assembly hall, leaving a space between the participants to avoid influence between classmates. After completing the questionnaires, the authors proceeded to carry out a conference on all the topics covered in the research in each of the classrooms, in which all the students’ doubts were resolved.

This research did not use restrictive criteria in the assessment of CPV. It was evaluated on a scale that measures how often participants engaged in aggressive behavior towards their parents. This aimed to take into account all the responses on the CPV scale, both high and low incidence.

### 2.5. Statistical Analyses

Analyses were performed based on structural equation modeling (SEM) with the IBM AMOS version 24 software (IBM SPSS, Chicago, IL, USA). The residual mean square error approximation (RMSEA) with a 90% confidence interval was used to examine the model fit. An RMSEA value ≤ 0.05 indicates adequate fit. The Tucker–Lewis index (TLI) and comparative fit index (CFI) were also applied. The minimum values of these indexes should be greater than 0.90, with values greater than 0.95 recommended for acceptance of the model [45]. To determine if the indirect effect through mediating variables was significant in predicting the dependent variable, we used the bootstrap method with 10,000 replications [46]. Other methods for demonstrating indirect effects assume a normal distribution to calculate p for the indirect effect, whereas the bootstrap method does not require the assumption of normality. We obtained the standard error and confidence interval for each of the model’s standardized coefficients. Participants who failed to complete all of the questionnaires were removed from the analyses. The minimum sample size for the model structure was calculated for a power level α = 0.95 (*p* < 0.05), taking into account the number of latent and observed variables, the recommended minimum size being 200 participants [47]. 

## 3. Results

First, the Mann–Whitney U-test compared the scores between the group of players and non-players in psychological, physical, and financial CPV against the mother and father. A non-parametric test was utilised because of the high skewness and kurtosis values found in some of the CPV variables. None of the differences between the two groups in the CPV scores was statistically significant. A Mann–Whitney U-test was also carried out comparing the scores between the group of violent video game players and non-violent game players in CPV against the mother and father. Lower scores on psychological violence towards both parents and on financial violence towards mothers were found in the violent video game players group.

Table 1 shows descriptive data on exposure to television violence, alcohol and drug use, engagement, and CPV (psychological, physical, and economic, against both parents) among video games players. We calculated the percentage of participants who committed serious assaults on their parents. Following the suggestion of Calvete et al. [44], the percentages of participants who reported threatening behavior, insults, blackmail, doing something to annoy their parents, disobeying an important order, or taking money without permission on more than six occasions were considered serious psychological or economic assaults. To assess severe physical aggression, the percentage of cases reporting physical assault on at least three to five occasions was taken into account. Thus, 26.83% acknowledged having committed serious psychological assaults against their mother and 22.50% against their father. In relation to severe physical aggression, 1.43% of the participants acknowledged that they had committed it against their mother, and 1.60% against their father. Finally, with regard to economic violence, 3.99% had committed it against their mother, and 2.89% against their father.

We then calculated the Pearson correlation matrix to verify the pattern of relationships and identify those that were excessive (*r* > 0.90; [41]), indicating multicollinearity. As can be seen in Table 2, multicollinearity was not a problem. The pattern of correlations was as expected, with a global relationship between exposure to television violence, alcohol and drug use, violent video game use, engagement, and the different types of violence against the mother and the father.

### 3.1. Initial Models

For the purpose of the study, the CPV variables (psychological, physical, and financial) were combined into the latent variable total CPV against the mother and total CPV against the father. Similarly, the variables of alcohol and drug use were combined into the latent variable substance use. Two structural equation models were calculated, first using the variable of total CPV against the mother and, subsequently, total CPV against the father.

The analysis was initiated with a model that included all the direct and indirect relationships of violent video game consumption with the total CPV, through the four variables of engagement. The effects of exposure to television violence and substance use were controlled, including the direct relationships of both variables with the total CPV. Finally, the participant’s sex was also controlled, including the relationship of sex with the variables of engagement and total CPV. Covariances among predictor variables were included in the model but are not presented for clarity. 

With regard to CPV against the mother, the model suggested that the consumption of violent video games was related to higher levels of immersion (β = 0.09, *p* < 0.05), presence (β = 0.12, *p* < 0.01), and flow (β = 0.11, *p* < 0.05). Flow was related to higher levels of total CPV against the mother (β = 0.21, *p* < 0.001). The direct relationship between violent video game consumption and the total CPV was also significant (β = −0.17, *p* < 0.001). 

On the other hand, the model also showed the existence of the direct relationship between exposure to violence on television and substance use and the total CPV against the mother (β = 0.15, *p* < 0.001, and β = 0.51, *p* < 0.001). However, the relationships of the consumption of violent video games with absorption, presence, immersion, and of absorption with the total CPV were nonsignificant. Similarly, relationships between the participant’s sex and the four variables of engagement and total CPV were also nonsignificant. 

The total CPV against the mother model’s fit index, χ^2^(28) = 37.80, *p* < 0.102, was as follows: RMSEA = 0.025 (90% CI (0.000, 0.044)), TLI = 0.981, and CFI = 0.992, within recommended limits [41]. The model obtained a predicted 35% of the variance of CPV against the mother.

With regard to CPV against the father, the model showed that higher rates of consumption of violent video games were related to higher levels of flow (β = 0.10, *p* < 0.05) and presence (β = 0.12, *p* < 0.01). Flow and absorption, on the other hand, were directly related to higher levels of total CPV against the father (β = 0.18, *p* < 0.001, and β = 0.15, *p* < 0.01, respectively). Finally, substance use was directly related to higher scores in total CPV (β = 0.48, *p* < 0.01).

However, the relationships between the consumption of violent video games and absorption and immersion, as well as the relationship between the latter two and presence with the total CPV were nonsignificant. Similarly, the relationship between the exposure to television violence and total CPV was also nonsignificant. Finally, relationships between the participant sex and the four variables of engagement and total CPV were also nonsignificant. 

The fit index of the model of violence against the father, χ^2^(28) = 41.70, *p* < 0.046, was as follows: RMSEA = 0.030 (90% CI (0.004, 0.047)), TLI = 0.973, and CFI = 0.989, also within the recommended limits [41]. In this case, the model obtained predicted 30% of the variance of the total CPV against the father.

The next goal was to determine the simplest model that fit the data in the best possible way. Concerning the model of the total CPV against the mother, the relationship between absorption and the consumption of violent video games and CPV were nonsignificant, so absorption was eliminated. Participant sex was also eliminated from the model. Finally, the relationships between presence and immersion with the total CPV were also excluded from the model because they were nonsignificant. Concerning the model of the total CPV against the father, the relationships between immersion and the consumption of violent video games and CPV were nonsignificant, so immersion was eliminated. The relationship between violent video game consumption and absorption and between presence and total CPV were also eliminated because they were nonsignificant. As in the model of violence against the mother, none of the relationships with sex were significant, so sex was eliminated. Finally, the variable of exposure to television violence was also eliminated, as its relationship with the total CPV was nonsignificant. 

### 3.2. Final Models

**Figure 1:**
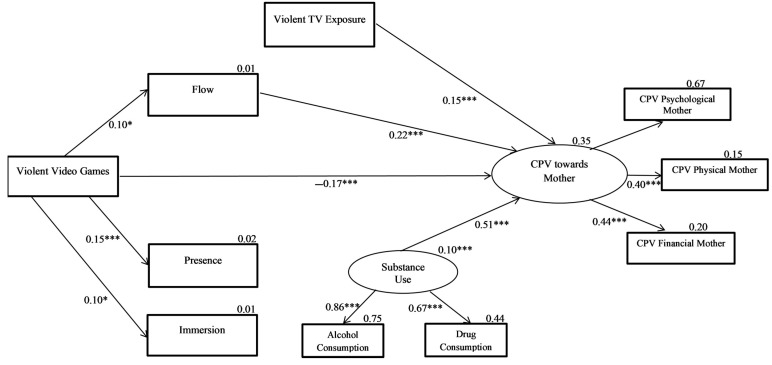
Predictive model of CPV towards mothers (final model). Rectangles represent observed variables and ovals represent latent variables. Values listed are standardized coefficients. * *p* < 0.05. *** *p* < 0.001.

Once the nonsignificant relationships were removed, the total CPV models against the mother and father were recalculated. The final model of violence against the mother is shown in Figure 1. Concerning the final fit index of the model, χ^2^(21) = 25.01, *p* < 0.247, the RMSEA improved slightly, reaching 0.018 (90% CI (0.000, 0.042)), the CFI increased to 0.996, and the TLI to 0.990. Table 3 shows the effects of the predictor variables on the total CPV against the mother in this final model. Removing the nonsignificant relationships did not reduce the strength of the remaining relationships of the model. The model obtained predicted 35% of the variance of total CPV against the mother.

**Figure 2:**
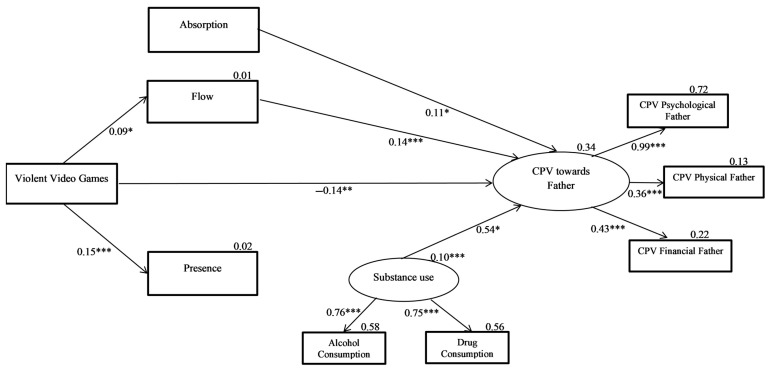
Predictive model of CPV towards fathers (final model). Rectangles represent observed variables and ovals represent latent variables. Values listed are standardized coefficients. * *p* < 0.05. ** *p* < 0.01 *** *p* < 0.001.

The final model of violence against the father is shown in Figure 2. Regarding the final fit index of the model, χ^2^(19) = 20.50, *p* < 0.365, the RMSEA was 0.012 (90% CI (0.000, 0.040)). The CFI and TLI indices increased to 0.998 and 0.997, respectively. Table 4 shows the effects of the predictor variables on the total CPV against the father in this model. As in the case of CPV against the mother, the elimination of the nonsignificant relationships did not reduce the strength of the remaining relationships of the model. This final model predicted 34% of the variance of CPV against the father.

## 4. Discussion

This study provides results that can contribute to understanding the phenomenon of CPV. The main objective of the investigation was to examine which variables could predict abuse against the parents. The mediating role of engagement (flow, immersion, absorption, and presence) was analyzed in the relationship between the consumption of violent video games and CPV against the mother and father. Variables of alcohol and drug use and exposure to television violence were considered control variables, along with the sex of the participants. The issue of whether the consumption of violent video games among adolescents leads to an increase in violent behavior is critical. In fact, our data indicate that these types of video games were played by almost half of the females and more than three-quarters of the males in our sample.

First, an analysis was carried out to compare the levels of CPV in young players and non-players. There were no statistically significant differences between them. These results are consistent with those from studies such as that of Kühn, Kugler, Schmalen, Weichenberger, Witt, and Gallinat [48], which found no differences between participants who had played violent video games, nonviolent video games, and non-players in terms of committing aggressive behaviors, impulsivity, empathy, or executive functions. However, in this study, lower scores on psychological violence towards both parents and financial violence towards mothers were found in the violent video games players group.

Then, focusing solely on the sample of young players, we analyzed the possible influence of violent video games on the CPV against the mother and the father. In both cases, and contrary to our hypothesis, we found an inversely proportional relationship, such that the greater the use of violent video games, the less the commission of violent behaviors against both parents. These results follow similar findings of authors such as Cunningham et al. [26], Jones [49], or Markey et al. [27], who confirmed the hypothesis of the existence of an inverse relationship between the consumption of violent video games and aggression and hostility. This inverse relationship may be caused by an effect of catharsis or incapacitation. It is possible that video games act as a release for aggression so that real expressions of aggression are decreased. It is also possible that, due to the time that playing video games takes, it draws players from other activities, including CPV perpetration.

However, it is important to note that some studies contradict our results, observing an increase in violence as a result of the use of violent video games [21,22]. However, so far, no study had analyzed the relationship between violent video games use and aggression in the context of CPV. 

As for the relationship between the consumption of violent video games and engagement, in agreement with our hypothesis, an effect of consumption was found on engagement, differing as a function of whether the violence was against the mother or the father. In the case of the mother, there was an influence of violent video games on the dimensions of flow, presence, and immersion. In the case of the father, an influence was observed on the dimensions of flow and presence. Although very few studies have empirically analyzed this aspect, Nowak et al. [34] found that players exposed to violent video games experienced more presence.

Concerning the relationship of engagement and CPV, in agreement with our hypothesis, the results show, on the one hand, a positive relationship between the flow dimension and CPV performed against both the mother and the father. On the other hand, an influence of absorption was found only on CPV against the father, increasing the levels of violent behavior. Taking into account the lower levels of CPV towards fathers found in this study, it is possible that this type of violence requires a more extreme level of engagement to take place (e.g., absorption). 

However, for the rest of the dimensions (presence and immersion), there was no significant relationship with CPV against the mother or the father. These results can be extrapolated to the conceptual model of Funk, Pasold, and Baumgardner [50], who argue that the flow and absorption caused by violent video games are moderating factors of aggressive behavior in adolescents, coinciding with the results found by authors such as Farrar et al. [38] and Nowak et al. [34]. The lack of a relationship between presence and immersion with CPV could be due to the fact that, whilst in the event of both flow and absorption, an altered state of consciousness is assumed, this does not occur during the states of presence and immersion. 

With regard to the influence of alcohol and drug use on CPV, it was shown to be a strong predictor of violence against both the mother and the father. These results are consistent with previous research (e.g., [12]), which states that drug use by minors is a common factor in cases of assaults on parents. Pereira [51] proposes that these consumptions are a risk factor for CPV because they diminish emotional control and also increase impulsivity to perform aggressive behaviors.

Regarding exposure to television violence, we found an influence of this variable only on CPV against the mother. This result is consistent with that obtained in studies such as that of Fitzpatrick et al. [40]. Authors such as Orue and Calvete [52], considering witnessing violence in four different contexts (home, neighborhood, school, and television), concluded that exposure to violence on television or at home predicts aggressive behavior, but they did not find this association in neighborhood or school contexts. However, Ferguson [24] found no link between the exposure to television violence and aggression in young people. 

Finally, concerning the last of the control variables, the sex of the participant, it should be noted that no relationship was found with any of the study variables. The findings of previous studies on the role of sex in engagement are contradictory. On the one hand, Nowak et al. [34] found that even though males played video games more, they experienced less presence than females. However, Farrar et al. [38] found that males became more involved in video games, regardless of the point of view used in the game.

One of the strengths of this study is that we did not ask participants for their subjective assessment of the level of violence of the video games they consumed. Asking for this as well as for their subjective assessment of their own violent behaviors could lead to the introduction of possible confounding factors. We minimized this possible bias by classifying the violent content of games using an independent, expertly crafted system, the PEGI.

This research presents some limitations that should be taken into account. The main limitation is the correlational nature of the study. Current findings should be replicated through longitudinal designs, which would allow us to examine the strength and directions of causal relationships. Another limitation concerns the number of adolescents committing severe physical and economic violence against their parents, as the percentages did not reach 5% of the sample. This must be taken into account when interpreting the results. However, the prevalence of severe physical assaults found in this study is virtually identical to that found by other investigations that used the same instrument to assess assaults on the parents [44]. With regard to control variables, in this study substance use and the exposure to violence on television were controlled in the structural equation models. However, there are other factors that were not controlled for in this research and have been identified as playing a role in CPV, such as peer violence or parental style.

Another concern relates to the response bias and adolescents’ willingness to disclose sensitive personal information. Finally, in this study, we make inferences about the effects of video games in general on the adolescent population as a whole. But specific groups of players who share factors associated with the use of technology, such as material deprivation, may be influenced differently by video games.

## 5. Conclusions

Despite its limitations, this study contributes to increasing knowledge about the characteristic psychological profiles of CPV. In this research, we found that the consumption of violent video games is strongly associated with lower CPV rates. However, although less relevant, it was also found that, to the extent that playing such video games provokes a sense of flow, it may counteract the protective role of violent video games against CPV. On the other hand, absorption, in the specific case of CPV against the father, may also be a causal factor of CPV. In conclusion, CPV was not related to video game consumption in general or specific violent video game playing, but to altered states of consciousness from engagement in the game. 

The results of this study, therefore, show the importance of taking these variables into account when preventing CPV, allowing us to take preventive measures in the face of these situations, such as by offering guidelines to schools and families on CPV prevention. Such guidelines can also be transferred to families by guidance teams of centers and those in charge of parenting schools to prevent future cases of violence. In this way, it would be possible to modify the specific habits of children in terms of the consumption of video games, as well as to help them manage the possible emotions and thoughts that may arise during the game sessions. Specifically, the present results suggest that interventions should be aimed to prevent or reduce the deeper engagement associated with an altered state of consciousness. Both flow and absorption consist of states of significantly altered consciousness, and deeply engaged players could experience desensitization to the effects of video game violence completely out of conscious awareness [2].

## Figures and Tables

**Table 1:** Descriptive statistics of exposure to violence on TV, substance use, video games engagement and child-to-parent violence (CPV) among video games players.

Variable		M	SD	Min.	Max.	Skewness	Kurtosis	*N*
Violent TV exposure		7.47	3.28	0	12	−0.61	−0.45	590
Alcohol consumption		0.45	0.94	0	5	2.64	7.67	625
Drugs consumption		0.24	0.95	0	5	4.17	16.56	626
Absorption		6.37	1.77	5	15	1.66	2.93	605
Flow		14.07	3.61	8	25	0.67	−0.11	600
Presence		7.58	2.1	4	12	0.17	−0.76	605
Immersion		1.87	0.86	1	3	0.25	−1.62	607
CPV Mother	Psychological	4.03	3.31	0	18	1.08	1.02	626
	Physical	0.14	0.59	0	7	5.86	44.37	626
	Financial	0.37	0.75	0	3	2.19	4.23	626
CPV Father	Psychological	3.54	3.25	0	18	1.25	1.72	622
	Physical	0.13	0.57	0	6	5.81	40.84	622
	Financial	0.3	0.68	0	3	2.46	5.71	622

**Table 2:** Correlations of all of the variables examined in the study among video games players.

Variable	1	2	3	4	5	6	7	8	9	10	11	12	13	14	15
1. Sex															
2. Violent TV exposure	0.120 **														
	(590)														
3. Alcohol	−0.00	0.07													
	(625)	(589)													
4. Drugs	0.00	0.05	0.55 ***												
	(626)	(590)	(625)												
5. Violent video games	−0.397 ***	−0.07	0.10 **	0.12 ***											
	(622)	(584)	(619)	(620)											
6. Absorption	0.08 *	0.10 *	−0.02	0.01	−0.01										
	(605)	(570)	(602)	(603)	(601)										
7. Flow	−0.02	0.14 ***	−0.04	0.04	0.08 *	0.51 ***									
	(600)	(566)	(597)	(598)	(596)	(598)									
8. Presence	−0.11 **	0.12 **	−0.05	−0.02	0.13 ***	0.45 ***	0.58 ***								
	(605)	(570)	(602)	(603)	(601)	(603)	(598)								
9. Immersion	−0.06	0.10 *	−0.06	0.00	0.09 *	0.34 ***	0.44 ***	0.44 ***							
	(607)	(572)	(604)	(605)	(603)	(605)	(600)	(605)							
10. Psychological CPVM	0.08 *	0.19 ***	0.13 ***	0.12 ***	−0.10 **	0.19 ***	0.24 ***	0.13 ***	0.06						
	(626)	(588)	(623)	(624)	(620)	(603)	(598)	(603)	(605)						
11. Physical CPVM	−0.00	0.02	0.13 ***	0.09 *	0.05	0.15 ***	0.13 ***	0.02	0.11 **	0.33 ***					
	(626)	(588)	(623)	(624)	(620)	(603)	(598)	(603)	(605)	(626)					
12. Financial CPVM	−0.02	0.11 **	0.19 ***	0.16 ***	0.03	0.09 *	0.11 **	0.10 **	0.05	0.35 ***	0.19 ***				
	(626)	(588)	(623)	(624)	(620)	(603)	(598)	(603)	(605)	(626)	(626)				
13. Psychological CPVF	0.02	0.14 ***	0.14 ***	0.16 ***	−0.08 *	0.19 ***	0.21 ***	0.21 ***	0.04	0.77 ***	0.27 ***	0.30 ***			
	(622)	(584)	(619)	(620)	(616)	(599)	(595)	(599)	(601)	(620)	(620)	(620)			
14. Physical CPVF	−0.01	−0.03	0.12 **	0.14 ***	0.04	0.14 ***	0.07	0.03	0.09 *	0.20 ***	0.77 ***	0.10 *	0.31 ***		
	(622)	(584)	(619)	(620)	(616)	(599)	(595)	(599)	(601)	(620)	(620)	(620)	(622)		
15. Financial CPVF	−0.00	0.12 **	0.21 ***	0.21 ***	0.01	0.09 *	0.08 *	0.01	0.06	0.33 ***	0.23 ***	0.75 ***	0.40 ***	0.16 ***	
	(622)	(584)	(619)	(620)	(616)	(599)	(595)	(599)	(601)	(620)	(620)	(620)	(622)	(622)	

Note. Values in brackets are *N*. * p < 0.05. ** *p* < 0.01. *** *p* < 0.001. CPVM: Child-to-parent violence towards mother. CPVF: Child-to-parent violence towards father.

**Table 3:** Standardized effects of exposition to violence in TV, substance use, violent video games and engagement on CPV towards mothers (final model).

	Standardized Effects		
	Indirect Effects	Direct Effects	Total Effects
Violent TV Exposure		0.15	0.15
Substances Use		0.51	0.51
Violent Video Games	0.02	−0.17	−0.15
Flow		0.22	0.22

**Table 4:** Standardized effects of substance use, violent video games and engagement on CPV towards fathers (final model).

	Standardized Effects		
	Indirect Effects	Direct Effects	Total Effects
Substances Use		0.54	0.54
Violent Video Games	0.01	−0.14	−0.13
Absorption		0.11	0.11
Flow		0.14	0.14

## Data Availability

Data available on request due to privacy restrictions.
